# Spatial distribution pattern of immune cells is associated with patient prognosis in colorectal cancer

**DOI:** 10.1186/s12967-024-05418-x

**Published:** 2024-07-01

**Authors:** Rongfang Shen, Ying Huang, Deyang Kong, Wenhui Ma, Jie Liu, Haizeng Zhang, Shujun Cheng, Lin Feng

**Affiliations:** 1 State Key Laboratory of Molecular Oncology, Department of Etiology and Carcinogenesis, National Cancer Center/National Clinical Research Center for Cancer/Cancer Hospital, Chinese Academy of Medical Sciences and Peking Union Medical College, No. 17 Panjiayuannanli, Chaoyang District, Beijing, 100021 China; 2 Department of Head and Neck Surgical Oncology, National Cancer Center/National Clinical Research Center for Cancer/Cancer Hospital, Chinese Academy of Medical Sciences and Peking Union Medical College, Beijing, China; 3 Department of Colorectal Surgery, State Key Lab of Molecular Oncology, National Cancer Center/National Clinical Research Center for Cancer/Cancer Hospital, Chinese Academy of Medical Sciences and Peking Union Medical College, No. 17 Panjiayuannanli, Chaoyang District, Beijing, 100021 China; 4 Department of General Surgery, Nanfang Hospital, Southern Medical University, Guangzhou, China

**Keywords:** Colorectal cancer, Multiplex immunohistochemistry staining, Immune spatial context, Distance to tumor cell, Prognosis

## Abstract

**Background:**

The spatial context of tumor-infiltrating immune cells (TIICs) is important in predicting colorectal cancer (CRC) patients’ clinical outcomes. However, the prognostic value of the TIIC spatial distribution is unknown. Thus, we aimed to investigate the association between TIICs in situ and patient prognosis in a large CRC sample.

**Methods:**

We implemented multiplex immunohistochemistry staining technology in 190 CRC samples to quantify 14 TIIC subgroups in situ. To delineate the spatial relationship of TIICs to tumor cells, tissue slides were segmented into tumor cell and microenvironment compartments based on image recognition technology, and the distance between immune and tumor cells was calculated by implementing the computational pipeline phenoptr.

**Results:**

MPO^+^ neutrophils and CD68^+^IDO1^+^ tumor-associated macrophages (TAMs) were enriched in the epithelial compartment, and myeloid lineage cells were located nearest to tumor cells. Except for CD68^+^CD163^+^ TAMs, other cells were all positively associated with favorable prognosis. The prognostic predictive power of TIICs was highly related to their distance to tumor cells. Unsupervised clustering analysis divided colorectal cancer into three subtypes with distinct prognostic outcomes, and correlation analysis revealed the synergy among B cells, CD68^+^IDO1^+^TAMs, and T lineage cells in producing an effective immune response.

**Conclusions:**

Our study suggests that the integration of spatial localization with TIIC abundance is important for comprehensive prognostic assessment.

**Supplementary Information:**

The online version contains supplementary material available at 10.1186/s12967-024-05418-x.

## Novelty and impact

Our study is the first to depict the spatial distribution pattern of TME cells of colorectal cancer in a large cohort. We highlighted that myeloid lineage cells were located closest to tumor cells and found that the prognostic value of T cells was closely related with its spatial distance to cancer cells.

## Background

Colorectal cancer (CRC) is a highly heterogeneous cancer and the second most deadly cancer worldwide [1]. However, patients with identical clinical features, such as TNM stage, often exhibit distinct outcomes. The pivotal role of the tumor microenvironment (TME) in CRC has been recognized, as patients with distinct immune and stromal cell infiltration patterns differ in clinical outcomes [2] and potential immunotherapy benefits [3]. However, current treatment regimens often neglect the unique composition of tumor-infiltrating immune cells (TIICs). A comprehensive understanding of the immune context in CRC is a prerequisite to resolving the treatment dilemma for these patients.

The immune context is the critical determinant of the clinical outcome in the management of CRC. The prognostic value of the immunoscore defined by the CD3^+^ and CD8^+^ T-cell infiltration pattern was shown to be superior to that of the TNM staging system^4^ and microsatellite instability status [5], promoting the establishment of an immune or TNM-immune classification system. Additionally, recent advances in single-cell RNA sequencing have demonstrated the comprehensive heterogeneity of the TME in CRC [6–8], which deepens our understanding of the role of TIICs in CRC tumorigenesis and cancer progression. In addition to T cells, the pivotal roles of tumor-associated macrophages (TAMs), cancer-associated fibroblasts (CAFs) [9], and B cells in controlling cancer progression by influencing T cells have recently been identified. Immune cells are spatially organized into a well-organized ecosystem [10], and their heterogeneity reflects the differences in different tumor niches [11]. However, few studies have focused on the spatial distribution of immune cells and evaluated the influence of the distance between a cancer cell and an immune cell.

Technologies such as spatially resolved transcriptomics [11] and imaging mass cytometry [12] capture cellular gene expression data in situ, providing massive genomic information and paving the way to investigate the TME structure of tumor niches. One apparent disadvantage of the abovementioned approaches is their high costs, thus limiting their widespread application to immune-oncology studies, clinical diagnosis, and treatment. In addition, the resolution cannot reach that of a single cell.

Multiplex immunohistochemistry (mIHC) staining utilizes tyramide signal amplification (TSA®) technology to simultaneously detect 7 markers on a single tissue slide. By incorporating the standardized image analysis workflow, mIHC can provide highly repeatable, stable, and cost-effective tissue and TIIC data [13, 14]. Here, we implemented mIHC to generate multidimensional immune spatial images of tumor center tissue cores from 190 enrolled CRC patients. We identified 14 TIIC phenotypes and compared the spatial distribution patterns between the epithelial and TME compartments in CRC tumors. In addition, the spatial impact on the antitumor effect was estimated by measuring the shortest distance between the TIICs to the surrounding cancer cells and finding that myeloid lineage cells were the closest to the cancer cells. Importantly, we found that the spatial location of immune cells was closely associated with their prognostic value, indicating that the spatial context of the immune cell structure needs to be considered in future studies.

## Materials and methods

### Patient enrollment and quality control

Two types of tissue microarray (TMA) were purchased from Shanghai Outdo Biotech Company (Shanghai, China) and used to analyze the samples from 100 patients with colon cancer (TMA ID: sur10) and 90 patients with rectal cancer (TMA ID: sur05). Clinicopathologic characteristics, including TNM staging system, age, sex, and overall survival of more than 5 years, were retrieved (Table 1, Table S1). Patients enrolled in this study did not receive chemotherapy or radiotherapy before surgery. The included TMA tissues were subjected to mIHC staining. TMA tissue cores without intact tissue structure or containing normal colorectal mucosa, high levels of necrosis or fibrosis, and unqualified tissue segments were excluded to ensure the quality of our data analysis.

**Table 1:** Clinical characteristics of enrolled patients

		sur05 (n = 90)	sur10 (n = 100)	P value
Cancer type (%)	Rectum cancer	Rectum cancer	Colon cancer	
Age (%)	< 65	41 (45.6)	38 (38.4)	0.395
	≥65	49 (54.4)	61 (61.6)	
Gender (%)	Female	42 (46.7)	41 (41.4)	0.562
	Male	48 (53.3)	58 (58.6)	
Location (%)	Right colon	0 (0.0)	50 (54.3)	<0.001
	Left colon	0 (0.0)	42 (45.7)	
	Rectum	90 (100.0)	0 (0.0)	
Maximum diameter (%)	< 5	45 (50.0)	36 (36.4)	0.081
	≥5	45 (50.0)	63 (63.6)	
T (%)	T1	2 (2.2)	0 (0.0)	<0.001
	T2	10 (11.2)	4 (4.0)	
	T3	76 (85.4)	64 (64.0)	
	T4	1 (1.1)	32 (32.0)	
N (%)	N0	54 (60.0)	52 (52.0)	0.384
	N1	24 (26.7)	36 (36.0)	
	N2	12 (13.3)	12 (12.0)	
M (%)	M0	89 (98.9)	95 (95.0)	0.265
	M1	1 (1.1)	5 (5.0)	
AJCC7 (%)	AJCC1	12 (13.3)	4 (4.0)	0.061
	AJCC2	41 (45.6)	47 (47.0)	
	AJCC3	36 (40.0)	44 (44.0)	
	AJCC4	1 (1.1)	5 (5.0)	
OS event (%)	0	49 (54.4)	48 (48.0)	0.458
	1	41 (45.6)	52 (52.0)	

### Multiplex immunohistochemistry

A PANO IHC kit (Panovue, China) was used for mIHC staining, which enabled the staining of multiple molecules on one formalin-fixed paraffin-embedded (FFPE) tissue slide simultaneously. The PANO IHC kit was applied following the manufacturer’s instructions with a detailed experimental procedure described previously [15]. Briefly, the TMA slide was subjected to deparaffinization, rehydration, antigen retrieval, antigen unmasking, and primary and secondary antibody incubation, similar to conventional immunohistochemistry staining. Fluorophore signaling linking to horseradish peroxidase (HRP) at the tissue site was generated by covalent bond binding. Multiple rounds of antigen retrieval steps involving heating, primary and secondary antibody incubation, and fluorescent dye staining were performed. Multiplex molecular staining in situ was achieved by retaining the fluorescent signal that was not removed by antigen retrieval each round. For the negative control, we implemented the above procedure but excluded the primary antibodies. Whole slide scanning and multispectral imaging at 20×magnification were completed by Vectra® Polaris™.

### Image analysis

Multiplex IHC images were analyzed with inForm software (version 2.4), incorporating spectral unmixing, tissue segmentation, cell segmentation, phenotyping, and scoring. We chose 4–5 tissue cores for analysis algorithm construction. During the tissue segment training process, at least 5 regions drew in each epithelial and TME tissue category, achieving a segmentation accuracy of 95%. Post cell nucleus identification, associated cytoplasm and membrane were discerned using default settings. For cell phenotyping training, we chose a minimum of 25 cells per phenotype as recommended by PerkinElmer. The finalized algorithm was employed in a batch analysis pipeline across all sampled tumor cores. Tissue segment quality was verified by manually checking the tissue segment images and their corresponding pathological images generated by autofluorescence. Tumor cores whose images could not be correctly segmented were excluded.

### H-score and cell density calculation

The fluorescence intensity of each molecular component was scored into 4 levels (0–3 score) with the fluorescence intensity of the positively stained cell used as a reference. For each tumor core image, the proportion of cells in the 4 score levels was calculated and used to then calculate the H-score:

H-score =100 x (1× Score 1% + 2 × Score 2% + 3 × Score 3%).

Score 1%, Score 2%, and Score 3% indicate the percentage of cells within scores 1, 2, and 3, respectively.

Cells classified into scores of 1–3 were considered molecular component-positive cells in the following analysis, and cell density was quantified as the number of positive cells per millimeter squared.

### Tumor purity

The tissue segment result of each image was saved in a file named cell_seg_data_summary.txt generated by inForm software (version 2.4). Tumor purity was calculated as the number of cancer cells divided by the number of cells in the whole tissue image.

### Immune cell-epithelium distance computation and effective cell definition

The distance between an immune cell and the tumor cells was defined as the distance between the immune cell and its nearest tumor cells. Cells in the epithelial compartment were considered tumor cells, and the nearest distance was calculated through the “find_nearest_distance” function wrapped in the “phenoptr” R package. Immune cells that had at least one tumor cell within a predefined radius were defined as effective immune cells and were inferred through the “count_within” function of the “phenoptr” R package (version 0.3.1).

### Unsupervised clustering

The unsupervised clustering was conducted by implemented the hierarchical clustering using the “pheatmap” function in “pheatmap” R package (version 1.0.12). The ward.D2 method was employed for data clustering and the optimal number of clusters was determined by the elbow method and silhouette scores. Heatmap plot with dendrogram was applied for visualization.

### Statistical analysis

All statistical analyses were performed using R (version 4.0.3). The Wilcoxon rank-sum test and Kruskal–Wallis rank sum test were adopted to compare data between two groups and more than two groups, respectively. P value < 0.05 was considered as statistical significance. The log-rank test was implemented to assess significant differences in survival analysis, and Kaplan‒Meier plots were used for visualization.

## Results

### Evaluation of TIIC biomarkers

**Fig. 1:**
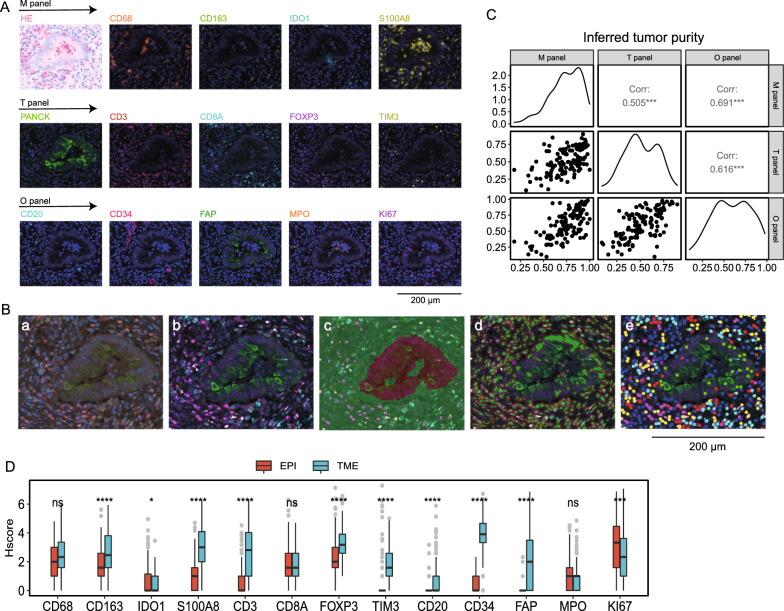
Study design overview and quality control. **A** Representative multiplex immunostained images of samples subjected to the 3 designed panels for the same patient. An autofluorescence-simulated HE histopathology image of the M panel is also shown. Scale bar, 200 μm. **B** Image processing workflow implemented by inForm software. The input image **a** was subjected to spectral unmixing **b**, tissue segmentation into epithelial and TME compartments (red: epithelium; green: TME) **c**, cell segments **d** and phenotyping **e**. **C** Correlation dot plot and density plot of the inferred purity. The correlation coefficient and p value are shown in the text. **D** H-score of the assessed molecules comparing the epithelial and TME compartments from the same patient. Bars show the median with interquartile range. The paired Wilcoxon test was applied, with ns > 0.05 *p < 0.05, **p < 0.01, ***p < 0.001, and ****p < 0.0001

To comprehensively evaluate the spatial distribution pattern of TIICs in colorectal cancer, we developed 3 immune cell marker panels, including macrophages, T cells, and B cells (Fig. 1A). Previously, we identified 3 TAM subtypes: TAM1, TAM2, and TAM3[16]. TAM1s express a specific marker of M2-type macrophages. TAM2s, which are said to attract and activate T cells, express the immune suppressive molecule indoleamine 2,3-dioxygenase 1 (*IDO1*) and interferon-related genes (*ISG15*, *ISG20*, *GBP1*). TAM3 is a proinflammatory macrophage, and its marker genes include *FCN1*, *S100A8*, *S100A9,* and *IL1B*. Thus, we incorporated our previously published macrophage cell marker panel (M-panel)[16] to evaluate the cooperative network between TAMs and other TME cells. The M-panel contains 4 myeloid lineage antibodies: *CD68* (pan macrophages), *CD163* (TAM1), *IDO1* (TAM2), and *S100A8* (TAM3). For the T-cell panel (T-panel), we chose *CD3* (pan T cells), *CD8A* (cytotoxic T cells), *FOXP3* (regulatory T cells), and *TIM3* (exhausted T cells) to indicate the functional status of T-cell subgroups and the epithelial cell marker gene *PANCK* for the accurate recognition and segmentation of the epithelial compartment. In addition, we also designed one cell panel, the “other” cell panel (O-panel), encompassing *FAP* (fibroblast), *CD20* (B cells), *Ki-67* (proliferative marker), *MPO* (neutrophil), and *CD34* (microvascular endothelial cell), to interactively analyze the major immune subtypes of CRC. The *FAP* staining intensity reflects the degree of cancer fibrosis. Most *Ki-67*-positive cells were cancer cells, which also aided in tissue segmentation. TMA arrays identical to the M-panel were subjected to the T- and O- panels. Detailed experimental procedures are provided in Methods section, and the staining schemes used in this study are given in Figure S1.

Then, the tumor tissues were divided into TME and epithelial compartments by implementing inForm software for image analysis (Fig. 1B). The cell nucleus staining marker DAPI allowed us to perform cell segmentation, which is the basis for cell phenotype assessment and scoring (Fig. 1B). To ensure the accuracy and repeatability of tissue staining and segmentation results, we compared tumor purity across the three panels and achieved high consistency (Fig. 1C). In total, 141, 164, and 160 of the M-, T-, and O-panels, respectively, passed the quality control (Figure S2). As expected, the vast majority of immune cell markers, such as *CD163*, *S100A8*, *CD3*, and *FOXP3*, showed higher expression levels in the TME compartment, while Ki-67, which was detected in proliferative tumor cells, was upregulated in tumor cells. Interestingly, we observed elevated expression levels of *IDO1* in tumor cells (Fig. 1D). In summary, our designed mIHC panel provided multiparametric information concerning the cellular location and staining intensity of each labeled protein, covering various kinds of immune cells previously identified in the single-cell immune landscape of CRC [6, 7, 17].

### Identification of TIIC phenotype and its distribution pattern

**Fig. 2:**
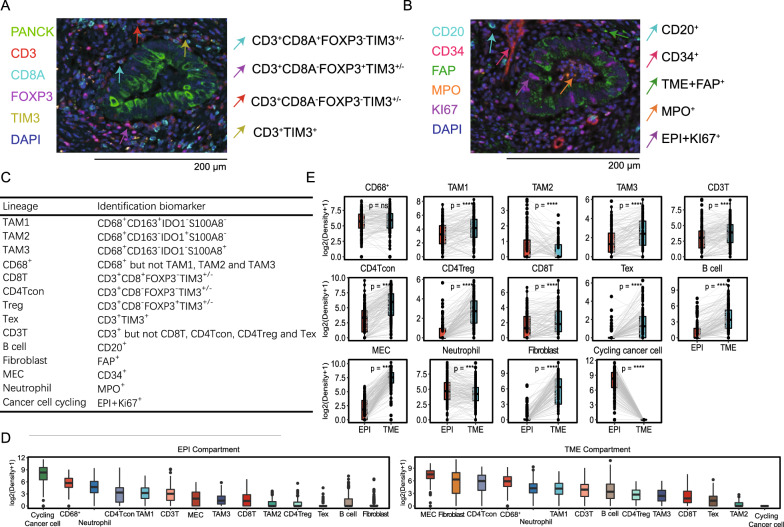
Multiplex IHC visualization and immune phenotype assignment. Colocalization of molecular markers identified immune cell phenotypes in the T-panel **A** and O-panel **B**. The corresponding single-color image of each molecular composite is shown in Fig. 1A. Scale bars, 200 μm. **C** Combined molecular markers for immune phenotype assignment. **D** Bar plot (median and interquartile range bar) shows the infiltration density. **E** Paired comparison between the density of the assigned immune cells in the epithelial and TME compartments, with n.s. > 0.05 and ****p < 0.0001

We next sought to identify TIIC subtypes based on mIHC data, which have been widely used to validate newly identified cell types discovered in single-cell studies. Here, we reported 4 TAM subtypes, 5 T-lineage subgroups, and 5 immune subtypes of the O-panel. TAM1s, TAM2s, and TAM3s were defined as *CD68*-positive cells simultaneously expressing *CD163*, *IDO1*, and *S100A8*, respectively; *CD68*^+^ cells were those that were positive only for *CD68*[16]. CD3 T cells (CD3T), CD8 T cells (CD8T), conventional CD4 T cells (CD4Tcon), Tregs and exhausted T cells (Tex) were recognized based on the combinations of canonical marker genes in the T-panel (Fig. 2A). Notably, we adopted the negative screening approach in the identification of the CD4Tcon, Tex, and CD3T phenotypes (Fig. 2A). *CD8A* and *TIM3* double-negative CD3^+^ cells were considered CD4Tcon cells, and Tex cells were those that were double-positive for *CD3* and *TIM3* after preferentially characterizing CD4Tcon cells. The same selection rule was applied to the CD3T and CD68^+^ phenotypes. For the O-panel, *CD20*, *FAP*, *CD34,* and *MPO* single-positive cells were deemed to be B cells, fibroblasts, microvascular endothelial cells (MECs), and neutrophils, respectively. Proliferative cancer cells were tumor cells positive for Ki-67 (Fig. 2B). The cellular phenotypes identified in the O-panel were exclusively single marker-positive cells. In total, we identified 14 phenotypes (Fig. 2C).

To determine the distribution pattern of TIICs, we first compared the infiltration level between the TME and epithelial compartments. MECs and fibroblasts were the most TME-enriched TIIC cells in CRC, while TAM2 had the smallest infiltration pattern (Fig. 2D). In contrast, myeloid lineage cells, including CD68^+^ cells, neutrophils and TAM1 cells, were highly dense in the epithelial compartment. When comparing the TIIC infiltration pattern between the tumor cell and TME compartments, we found that neutrophils and TAM2s exhibited higher infiltration levels in or close to the tumor parenchyma (Fig. 2E). In contrast, T lineages, B cells, TAM1s, TAM3s, and MECs were highly enriched in the TME compartment, as expected (Fig. 2E). Correlation analysis showed that B cells in tumor mass was enriched in early tumor (T) and AJCC stage (Figure S3A-B) and B cells in TME was associated with early T stage (Figure S3C), suggesting a protective role of B cells for limiting CRC staging. While the TAM3s of tumor mass was enriched in non-metastatic tumors (Figure S3D). When focused on the tumor location, the right-sided CRC showed increased level of fibroblasts and CD68^+^ cells, and reduce in the neutrophil population (Figure S3E-H).

Collectively, we identified 14 TIIC phenotypes using the mIHC, and revealed distinct distribution patterns. T, B and stromal lineages were located primarily in the TME compartments, while a higher density of neutrophils and TAM2 cells was observed in the epithelial compartment.

### Myeloid lineages located close to cancer cells

**Fig. 3:**
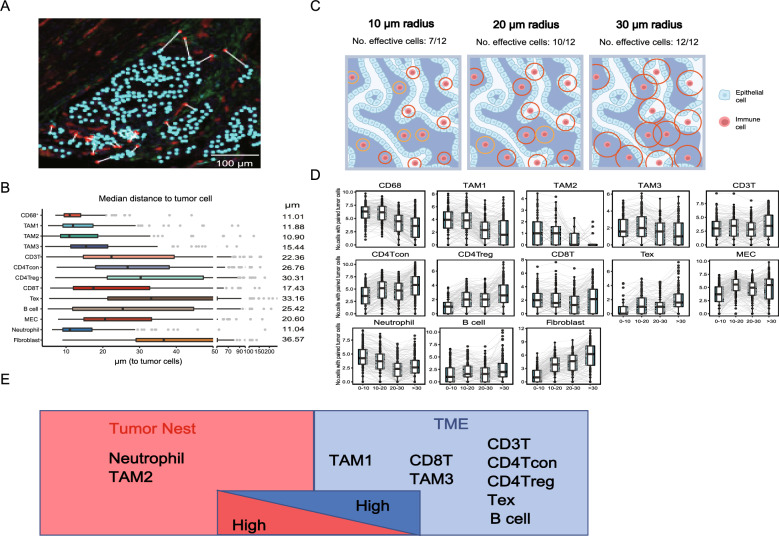
Distance analysis between immune cells and tumor cells. **A** A schematic diagram showing the definition of nearest distance, which is the distance between a TME cell and its nearest neighboring cancer cell. Scale bars, 100 μm. **B** Bar plot of the distribution of the nearest distance of the TME cells. Each dot represents a tumor sample, and the median value of the nearest distance is calculated. **C** Schematic figure illustrating the notation of effective immune cells within different preset radii. **D** Box plot summarizing the effective cell count within a 0–10 μm, 10–20 μm, 20–30 μm, and > 30 μm radius. **E** Schematic model of immune cell abundance from the tumor mass to the surrounding TME

To further investigate the spatial relationship between TIICs and cancer cells, we then calculated the distance between the TIICs and their nearest tumor cell (Fig. 3A). Similarly, myeloid lineages were found to have the closest spatial proximity to cancer cells (Fig. 3B). TAM2 was the nearest cell type, with a median distance of 10.90 μm, and TAM3 was the farthest cell type, with a 15.44 μm distance. Median distances of more than 20 μm were observed in the remaining TME cells, except for CD8 T cells, which had a median distance of 17.43 μm (Fig. 3B). Similarly, B cells showed shorter median distance in early T stage. In terms of tumor location, CD3T cells and neutrophil of the left-sided CRC exhibited more closer to cancer cells (Figure S4A–C).

To simultaneously take the number of TIICs and their proximity to cancer cells into consideration, we introduced the notion of effective immune cells (Fig. 3C). TIICs with at least one cancer cell within a preset radius were considered effective cells. Here, we recorded the number of immune cells within a 10, 20, 30 μm and over 30 μm radius as previously reported [18, 19] (Fig. 3D). Consistent with the spatial distribution pattern summarized in Fig. 3C, the majority of TAM1s (72.11%), TAM2s (85.92%), TAM3s (63.48%), CD68^+^cells (76.13%) and neutrophils (77.11%) had cancer cells located within 20 μm. In addition, the greatest number of effective neutrophils and TAM2 were identified within a 10 μm radius of a cancer cell (Fig. 3D).

Among all TIICs except those of myeloid lineages, B cells constituted a small fraction. In the 160 CRC patients who passed O-panel quality control, 8768 B cells were identified, with a mean of 54.8 cells per sample. The B cells were distributed evenly around cancer tissue. The majority of T cells did not have tumor cells within a 30 μm radius, which was particularly apparent for CD3^+^ T, CD4Tcon, Treg, and Tex cells. For the stromal lineage cells, we observed that the greatest number of MECs were found within 10–20 μm and over a 30 μm radius of a tumor cell. In contrast, FAP^+^ fibroblasts were least frequent at tumor zone and increased in count closer to the TME compartment (Fig. 3D). Taking the above observations together, a glimpse of the TIIC spatial landscape from the perspective of the proximity to tumor cells was proposed (Fig. 3E). Compared with T cells and B cells, myeloid lineage cells were closest to tumor cells. The spatial distance limit between cancer cells and T cells or B cells might hint at another immune escape mechanism in colorectal cancer.

### Proximity to tumor cells is associated with prognosis

**Fig. 4:**
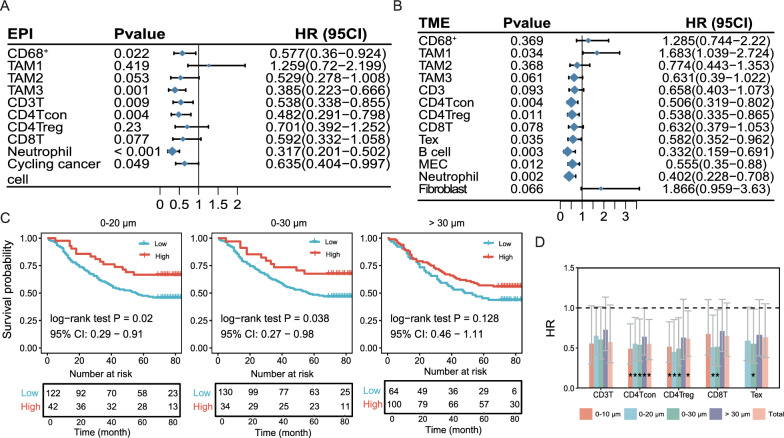
Overall survival analysis of the identified immune cells. Forest plot of immune cells in the epithelial compartment **A** and TME compartment **B**. P value, hazard ratio (HR), and 95% CI estimated by the Cox proportional hazards regression model. **C**. Kaplan‒Meier estimates of overall survival according to the number of effective cells within a 0–20 μm, 0–30 μm, and > 30 μm radius. **D**. Bar plot of the HR value and 95% CI, with bar height representing the HR value. Error bar, ± 95% CI. P values less than 0.05 are indicated with a star

Given that the level of infiltration and spatial distribution of TIICs exhibited a close relationship with CRC patient prognosis, we next sought to conduct a survival analysis of TIICs in the context of spatial location. Epithelial compartment-enriched TAM2s and neutrophils exhibited a favorable prognostic effect, although that for the TAM2s did not reach statistical significance (p=0.053) (Fig. 4A). Tex, B cells, MECs, and fibroblasts were excluded from the assessment in the epithelial compartment owing to their negligible amounts. In the TME, the infiltration level of TAM1 and the tissue fibrosis indicator, FAP^+^ fibroblasts, were unfavorable prognostic factors (Fig. 4B). Similar to previous results, most infiltrating immune cells, including Tregs [20] and neutrophils [21], showed a positive impact on patient survival (Fig. 4A, 4B), emphasizing the key role of immune infiltration patterns in monitoring CRC patient survival. Except for cycling cancer cell and TAM1 in TME, the above statistically significant cells remain notably associated with prognosis after adjusting for age, gender and etc. (Table 2 ).

**Table 2:** Multivariable cox regression results of the identical cells

Cell	P	HR	95CI
EPI.CD3T	0.001	0.42	0.251–0.704
EPI.CD4Tcon	0.008	0.49	0.29–0.83
EPI.CD68^+^	0.013	0.527	0.317–0.876
Cycling cancer cell	0.056	0.629	0.391–1.011
EPI.Neutrophil	< 0.001	0.349	0.213–0.573
EPI.TAM3	0.001	0.376	0.208–0.679
TME.B cell	0.007	0.358	0.171–0.751
TME.MEC	0.011	0.538	0.333–0.867
TME.CD4Tcon	0.006	0.485	0.29–0.81
TME.CD4Treg	0.036	0.586	0.356–0.965
TME.Neutrophil	0.008	0.458	0.257–0.816
TME.TAM1	0.079	1.573	0.95–2.606
TME.Tex	0.01	0.488	0.283–0.841

Functional T cells must be located close to or in direct contact with tumor cells to exert an antitumor immune response. CD8T cells are collectively a well-acknowledged immune index that is closely related to greater survival [20, 22]. However, univariate Cox regression analysis in the epithelial and TME compartments showed that patients with higher quantities of CD8T cells tended to have better survival outcomes, this tendency was not significant. Intriguingly, we found that CD8T-cell infiltration levels within a 20 or 30 μm radius showed good prognostic power but not for those beyond a radius of 30 μm (Fig. 4C). CD8T tumor cells within a 15–20 μm radius allowed the immune cell to attack the tumor cells with which they were in direct contact, indicating that CD8T cells are effective when near tumor cells [15, 23]. Furthermore, no significant prognostic value was observed for Tregs, CD8Ts, and Texs located beyond a 30 μm radius (Fig. 4D). By taking the effective cells as measurement metrics, such as T-panel cells, the prognostic influence of M-panel and O-panel cells was evaluated (Figure S5). Notably, TAM1s located beyond a 30 μm radius was significantly unfavorable, whereas other preset distance ranges were not (Figure S5A). TAM1s exert their tumor growth-supporting role by influencing the function of T cells [24, 25], which might explain why the infiltration level of CD8 T cells located beyond 30 μm failed to predict the survival benefit for CRC patients. The comprehensive evaluation of the prognostic influence of effective immune cells emphasizes the importance of the spatial location of immune cells. The CD8T-mediated tumor-killing role was associated with its proximity to cancer cells, and the interaction with TAM1s beyond a 30 μm distance further attenuated the tumor-killing effect.

### B-cell and TAM2s are associated with improved overall survival

**Fig. 5:**
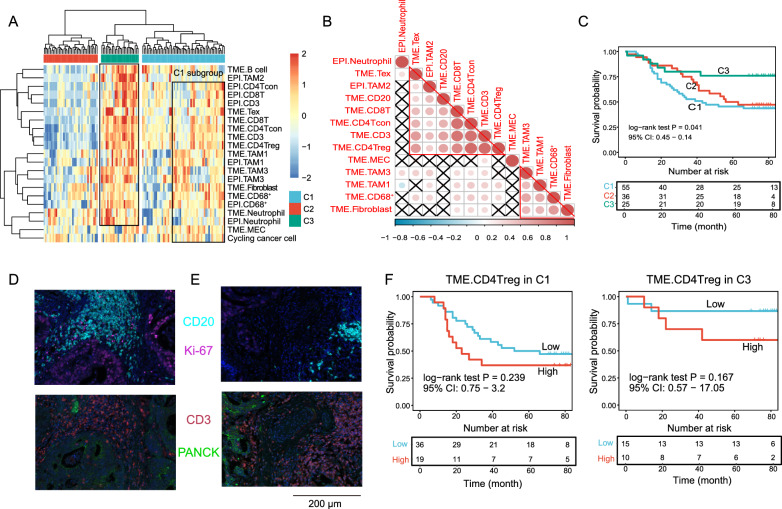
Immune subtype identification. **A** Unsupervised clustering of immune cells clustered the CRC samples into 3 subtypes. The **C3** and **C1** subgroups are highlighted with a rectangle. **B** Heatmap showing the correlation of the immune phenotypes. **C** KM plot for OS-related survival of the identified immune clusters. **D-E** Representative CD20- and CD3-stained images show the lymphoid follicles in the tumor mass **D** and invasive front (**E**). Ki-67 and PANCK are shown to aid in the identification of cancer cells. **F** Overall survival plot for the TME compartment Tregs in **C1** and **C3**

Considering that immune cells in the microenvironment are widely organized with cells infiltrated in a synergistic or exclusive pattern, we then wondered how these distinct tissue compartment-located immune cells cooperate in CRC to influence patient prognosis. Unsupervised classification of these identified TIICs revealed 3 tumor subclasses with distinct infiltration patterns. The clinical characteristics of these 3 subclasses are shown in Table 3. C3, C1, and C2 were considered highly, moderately, and poorly infiltrated subtypes, respectively (Fig. 5A). Immune cells showed a similar infiltration pattern between epithelial and TME compartments, indicating that immune cells in the epithelial compartment might migrate from the surrounding microenvironment (Fig. 5A). Correlation analysis also revealed two distinct immune clusters: myeloid-stromal lineage clusters and T-B clusters (Fig. 5B). As expected, the most highly infiltrated subtype, C3, exhibited the best overall survival. Unexpectedly, the most poorly infiltrated subtype, C2, was associated with better survival than the moderately infiltrated subtype, C1 (Fig. 5C). Further investigation indicated that C1 was highly heterogeneous, with one C1 subgroup showing a high infiltration pattern (Fig. 5A). Similarly, the C1 subgroup was associated with inferior survival (Figure S6A), and differential analysis indicated that B cells, neutrophils, CD8 T cells and TAM2 cells were the most evidently upregulated cells in C3 (Figure S6B). These results showed that TAM2s and B cells are necessary for T cells to attack tumor cells. The tumor-killing effect of T cells might be inhibited by TAM1s in the absence of TAM2s and B cells. Our previous study showed that TAM2s highly expressed CXCL9, CXCL10, CXCL11 and upregulated interferon-related genes, which are thought to play a role in causing T-cell infiltration [16, 26]. B cells, particularly those with a well-organized tertiary lymphoid structure (TLS), are widely known to be related to isotype switching and somatic hypermutation of the B-cell maturation process and tumor-specific T-cell responses [27, 28]. Correspondingly, immune cell correlation analysis suggested that TAM2s and B cells are strongly related to T lineage cells (Fig. 5B). Therefore, B cells and TAM2s appear to guarantee the activation and effector of T cells. Here, we found that 4 colon tumors and 3 rectum tumors presented with B-cell aggregation. However, only one identified B-cell aggregation structure resided solely in the tumor mass (Fig. 5D), with the majority located in the tumor surrounding stroma (Fig. 5E). In addition, all B-cell follicles detected in our study were in the early stage, lacking the T-cell zone. Nevertheless, patients with TLS exhibited superior outcomes. The patient with TLS who died 33 months postdiagnosis presented with distant metastasis. For the remaining patients, which were classified into stages II and III, all survived more than 5 years (Table S2).

**Table 3:** Patients’ clinical characteristics across the identified immune subtypes

		C1 (n = 55)	C2 (n = 36)	C3 (n = 25)	P value
Array (%)	Rectum cancer	19 (34.5)	23 (63.9)	13 (52.0)	0.02
	Colon cancer	36 (65.5)	13 (36.1)	12 (48.0)	
Age (%)	< 65	22 (40.7)	14 (38.9)	13 (52.0)	0.554
	≥65	32 (59.3)	22 (61.1)	12 (48.0)	
Gender (%)	Female	26 (48.1)	14 (38.9)	14 (56.0)	0.408
	Male	28 (51.9)	22 (61.1)	11 (44.0)	
Location (%)	Right colon	20 (37.7)	6 (17.1)	4 (16.7)	0.048
	Left colon	14 (26.4)	6 (17.1)	7 (29.2)	
	Rectum	19 (35.8)	23 (65.7)	13 (54.2)	
Size (%)	≥5	28 (50.9)	20 (55.6)	12 (50.0)	0.884
	< 5	27 (49.1)	16 (44.4)	12 (50.0)	
T (%)	T1	0 (0.0)	1 (2.8)	0 (0.0)	0.726
	T2	4 (7.3)	2 (5.6)	3 (12.0)	
	T3	42 (76.4)	29 (80.6)	18 (72.0)	
	T4	9 (16.4)	4 (11.1)	4 (16.0)	
N (%)	N0	32 (58.2)	23 (63.9)	15 (60.0)	0.542
	N1	21 (38.2)	9 (25.0)	8 (32.0)	
	N2	2 (3.6)	4 (11.1)	2 (8.0)	
M (%)	M0	52 (94.5)	34 (94.4)	25 (100.0)	0.488
	M1	3 (5.5)	2 (5.6)	0 (0.0)	
AJCC7 (%)	AJCC1	4 (7.3)	3 (8.3)	3 (12.0)	0.926
	AJCC2	28 (50.9)	18 (50.0)	12 (48.0)	
	AJCC3	20 (36.4)	13 (36.1)	10 (40.0)	
	AJCC4	3 (5.5)	2 (5.6)	0 (0.0)

The favorable prognostic effect of Tregs in CRC has been implied in several studies [20, 29], while others have reported that Tregs promote CRC progression by suppressing the CD4^+^T-cell response to tumor-associated antigens [30]. The underlying mechanism remains still unclear. We were surprised to find that Tregs were closely correlated with the remaining T lineage cells (Fig. 5B) and enriched in C3 and C1 (Fig. 5A). Survival analysis of Tregs in C3 and C1 demonstrated that they tended to be associated with worse survival in both subtypes (Fig. 5F). Accordingly, we speculated that the favorable prognostic value of Tregs was caused by the bias produced by the remaining T lineage cells.

## Discussion

Our study revealed the spatial distribution pattern of TIICs in colorectal cancer using mIHC technology. Evaluating the nearest distance between the cancer cells and TIICs, we found that TIICs were distributed unevenly and its spatial distribution was closely related with patient’s prognosis. The underlying reason why TIICs’ spatial location associated with CRC prognosis was to a great extent related to tumor heterogeneity. The most obvious evidence is that the microsatellite instability-high (MSI-H) CRC patients, in comparison with microsatellite stable (MSS) CRCs, harvested high infiltration level of CXCL13^+^ T cell. This CXCL13^+^ T enrichment may be associated with the high interferon-gamma (IFNγ) milieu characteristic of MSI-H tumors, which induces epithelial cells to express higher levels of ISGs such as CXCL9, CXCL10, and CXCL11, thereby recruiting activated T cells. Furthermore, CXCL10/CXCL11^+^ malignant cells are, on average, positioned closer to CXCL13^+^ T cells than their negative counterparts [10]. This phenomenon likely contributes to the superior prognosis and enhanced response to immunotherapy observed in MSI-H CRC patients.

Tumor cells utilize glycolysis to generate sufficient ATP for the rapid proliferation and unlimited replication of cancer cells. This metabolic mechanism simultaneously creates a surrounding microenvironment featuring low glucose levels, a low pH, and hypoxia, which are not comfortable for the survival of effector T cells but are suitable for suppressive myeloid cells such as myeloid-derived suppressor cells and TAMs [31]. Supporting this notion, we found that myeloid lineage cells were closest to tumor cells compared to T lineage cells and B cells. Thus, the spatial proximity of myeloid cells to tumor cells provides the essential prerequisite for the protection or eradication of tumors. However, the T lineage cells assessed in this study were located farther than 15 μm from a tumor cell, a distance beyond the cell‒cell direct contact distance proposed by a previous study [15]. Indirect contact of T cells with tumor cells might hint at another novel immune escape mechanism in colorectal cancer. These data suggest that distinct immune lineages had specialized distribution patterns in colorectal cancer and might be related to the metabolic activities of tumor cells.

A previous study reported that IDO1, the most common IDO family gene in CRC, was expressed in epithelial cells and myeloid cells but rarely in lymphoid cells [32]. IDO1 can catalyze tryptophan into suppressive kynurenine, which could promote Treg differentiation and induce PD1 expression in CD8 T cells [33]. In addition, IFNs can enhance their expression in DCs and macrophages, leading to immune tolerance [32]. These data indicated that IDO1 plays an immune-suppressive role in CRC and that its high expression in colon cancer cells is associated with adverse prognosis [34]. However, we found that the expression of IDO1 in macrophages, that is, TAM2s, was a good prognostic factor in our study. Correlation analysis strongly indicated that TAM2s were closely related to T lineage cells and especially B cells. Our data suggest that TAM2s might be related to active humoral immune defense or the functional work of TLSs, activating the immune response, and that *IDO1* might be a promising target for CRC immunotherapy.

Currently, the tumor-infiltrating T-cell level and its spatial location are considered strong prognostic indicators in CRC. Only T cells infiltrating the tumor mass can be mobilized to attack and eliminate tumor cells, but few studies have assessed the influence of the distance from T cells to tumor cells. Our further investigations indicated that the distance between immune cells and tumor cells was associated with patient prognosis. Assessment of the CD8T cell level within a 20 or 30 μm radius may be more appropriate for predicting patient prognosis. Other than CD8 T cells, previous studies have found that memory T cells, Th1 cells, and tissue-resident memory T cells were also associated with patient prognosis [35–37]. Furthermore, the prognostic impact of Tregs and Texs was more evident within 30 μm of a tumor cell, emphasizing the need for incorporating T-cell distance into considerations of the patient outcome. Therefore, incorporation of the T-cell’s spatial location with the number of infiltrating cells will help improve prognostic precision in further studies.

Neutrophils are short-lived, terminally differentiated, and non-proliferative cells that can be detected in 80–90% of CRC patients. As the key element affecting tumor inflammation, neutrophils can initiate DNA damage in epithelial cells by releasing reactive oxygen species, thus contributing to tumor onset [38]. Moreover, neutrophils can release neutrophil extracellular traps to protect cancer cells from killing by immune cells. For most cancers, neutrophils are associated with adverse survival [39], whereas their prognostic role in CRC is controversial, as conflicting outcomes have been reported [40, 41]. Our previous study showed that high levels of MPO^+^ neutrophils evaluated by IHC were related to adverse clinical outcomes in COAD [39]. However, MPO^+^ neutrophils were a strong indicator of superior prognosis in this study using the mIHC assay. Why the opposite results were observed in CRC is unknown. Recently, one study noted that neutrophils suppressed early-stage tumor growth but promoted late tumor outgrowth by influencing the homologous or nonhomologous recombination repair mechanism [42]. The presence of the microbiota in CRC further augments the difficulty in ascertaining the function of neutrophils [43]. The mechanism by which neutrophils change tumor ecology should be investigated in further studies.

In summary, our study revealed that myeloid lineage cells are located closest to tumor cells and that the spatial location or organized structure of TIICs influence patient prognosis. The mechanism underlying this distribution pattern may be associated with the metabolic features of cancer cells. In addition, the classification of immune subtypes according to the mIHC qualified immune abundance highlights the critical role of the cooperation between B cells and T cells in the prediction of a patient’s prognosis. Nevertheless, this study had some limitations. Due to the technical limitations of mIHC, we only assessed a maximum of 5 molecular subtypes to avoid the problem of color cross-talk. Thus, the immune cell spatial resolution was insufficient for discovering or evaluating the spatial impact of novel immune subtypes. Second, this was an exploratory study, and these observations require confirmation in multicenter, prospective studies.

### Supplementary Information

Additional file 1 Figure S1. mIHC experimental scheme used in this study. Note: The “order” column indicates the staining order of the identified molecule in each staining panel

Additional file 2 Figure S2. Flow chart of the inclusion and exclusion criteria. Red, blue, and yellow boxes represent the M-panel, T-panel, and O-panel, respectively. The number of excluded samples is shown in the purple box, and the number of samples common to all three panels is shown in the green box

Additional file 3 Figure S3. Density of TILCs across distinct tumor stages and locations. (**A**) Box plot depicting EPI.B cells infiltration level grouped by AJCC stages. (**B-C**) Density of EPI.B cells (**B**) and TME.B cells (**C**) in distinct T stages. (**D**) Density of EPI.TAM3 grouped by tumor. Metastasis. (**E-H**) Distribution of TME.Fibroblast (**E**), EPI.Fibroblast (**F**), EPI.Neutrophil (**G**) and TME.CD68^+^(**H**) cells in left-sided and right-sided CRC. Kruskal-Wallis and Wilcoxon p values are annotated by text

Additional file 4 Figure S4 Distribution of B cell (**A**), CD3T cell (**B**) and neutrophil cell (**C**) according to T stages (**A**) and tumor location (**B-C**). The Y-axis represents the nearest distance to cancer cells

Additional file 5 Figure S5. Bar plot showing the HR result for the M-panel–(**A**) and O-panel–(**B**) derived immune phenotypes. Bar height represents the HR value. Error bar, ± 95% CI. P values less than 0.05 are indicated with a star

Additional file 6 Figure S6. Survival and immune cell differences between the **C3** and **C1** subgroups. (**A**) Kaplan‒Meier analysis of overall survival between the **C3** and **C1** subgroups. P values and 95% CIs were calculated by the log-rank test. (**B**) The volcano plot illustrates the difference in immune cells between the **C3** and **C1** subgroups, with the absolute value of log2-fold change > 1 and p value < 0.05 as the cut point

Additional file 7

## Data Availability

Processed data could be found on Zenodo (https://zenodo.org/records/11211500) and the raw mIHC images of this study are available from the corresponding author upon reasonable request.

## Abbreviations

CAF: Cancer-associated fibroblast
CRC: Colorectal cancer
IDO1: Indoleamine 2,3-dioxygenase 1
MEC: Microvascular endothelial cell
mIHC: Multiplex immunohistochemistry
M-panel: Macrophage cell marker panel
MSI-H: Microsatellite instability-high
MSS: Microsatellite stable
O-panel: Other cell panel
IFNγ: Interferon-gamma
TAM: Tumor-associated macrophage
TIIC: Tumor-infiltrating immune cell
TLS: Tertiary lymphoid structure
TME: Tumor microenvironment
T-panel: T-cell panel
