# Supplementary material for: Spatial distribution pattern of immune cells is associated with patient prognosis in colorectal cancer
Source: J Transl Med. 2024 Jul 1;22:606. doi: 10.1186/s12967-024-05418-x (PMC11218284; doi:10.1186/s12967-024-05418-x)
Supplement: Supplementary file 1 — Additional file 1 Figure S1. mIHC experimental scheme used in this study. Note: The “order” column indicates the staining order of the identified molecule in each staining panel [file 12967_2024_5418_MOESM1_ESM.pdf]

M-panel

Antibody	Supplier	Catalog #	Dilution factor	AR buffer	Fluorophore	Order
CD163	CST	93498	1:400	citrate, PH = 6	520	1
IDO1	abcam	ab211017	1:3000	Tris-EDTA, PH = 9	540	2
S100A8	Proteintech	66853-1-Ig	1:1500	citrate, PH = 6	570	3
CD68	CST	76437	1:1000	citrate, PH = 6	650	4

T-panel

Antibody	Supplier	Catalog #	Dilution factor	AR buffer	Fluorophore	Order
PANCK	abcam	ab7753	1:2000	citrate, PH = 6	520	1
CD8A	CST	70306	1:200	Tris-EDTA, PH = 9	540	2
TIM3	CST	45208	1:200	Tris-EDTA, PH = 9	570	3
FOXP3	abcam	ab20034	1:200	citrate, PH = 6	620	4
CD3	proteintech	60181-1-Ig	1:4000	citrate, PH = 6	690	5

O-panel

Antibody	Supplier	Catalog #	Dilution factor	AR buffer	Fluorophore	Order
FAP	abcam	ab207178	1:100	citrate, PH = 6	520	1
CD20	CST	48750	1:200	citrate, PH = 6	540	2
KI67	zhong shan -golden	ZM-0166	1:20	citrate, PH = 6	620	3
MPO	CST	14569	1:2000	citrate, PH = 6	650	4
CD34	abcam	ab81289	1:2000	Tris-EDTA, PH = 9	690	5
