# Supplementary figures and images for: Spatial distribution pattern of immune cells is associated with patient prognosis in colorectal cancer
Source: J Transl Med. 2024 Jul 1;22:606. doi: 10.1186/s12967-024-05418-x (PMC11218284; doi:10.1186/s12967-024-05418-x)

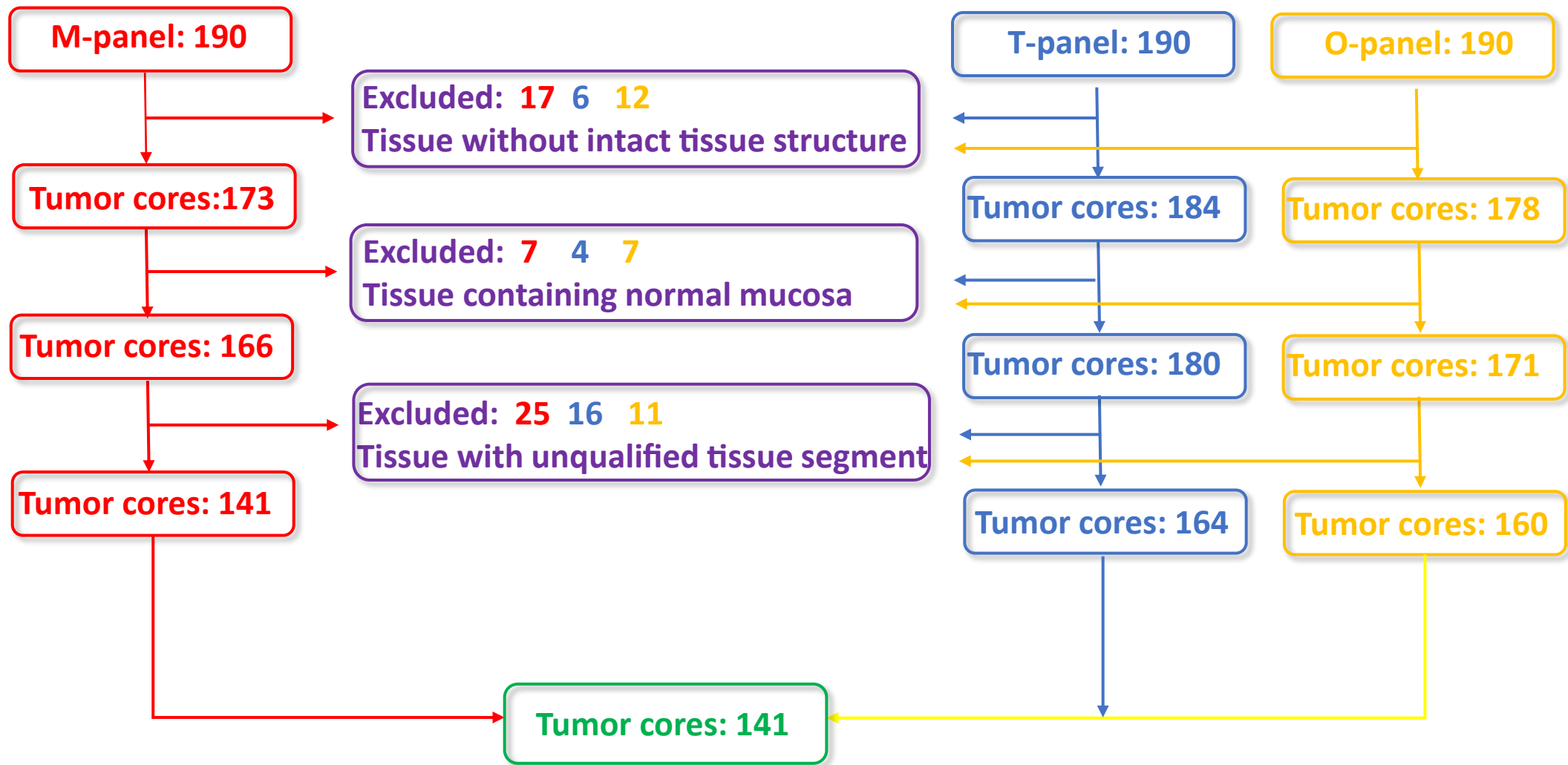

Supplement: Supplementary file 2 — Additional file 2 Figure S2. Flow chart of the inclusion and exclusion criteria. Red, blue, and yellow boxes represent the M-panel, T-panel, and O-panel, respectively. The number of excluded samples is shown in the purple box, and the number of samples common to all three panels is shown in the green box [file 12967_2024_5418_MOESM2_ESM.pdf]

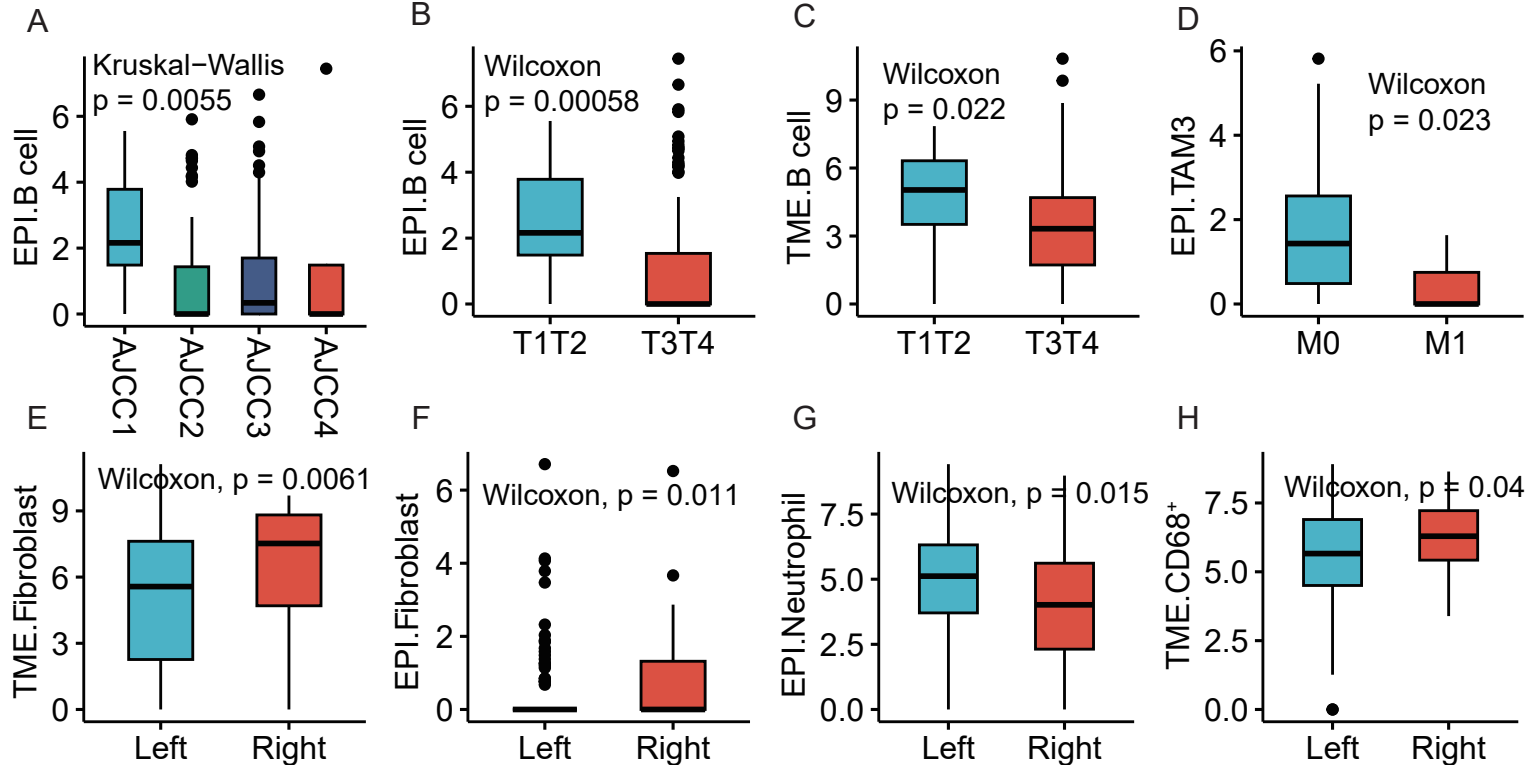

Supplement: Supplementary file 3 — Additional file 3 Figure S3. Density of TILCs across distinct tumor stages and locations. (A) Box plot depicting EPI.B cells infiltration level grouped by AJCC stages. (B-C) Density of EPI.B cells (B) and TME.B cells (C) in distinct T stages. (D) Density of EPI.TAM3 grouped by tumor. Metastasis. (E-H) Distribution of TME.Fibroblast (E), EPI.Fibroblast (F), EPI.Neutrophil (G) and TME.CD68+(H) cells in left-sided and right-sided CRC. Kruskal-Wallis and Wilcoxon p values are annotated by text [file 12967_2024_5418_MOESM3_ESM.pdf]

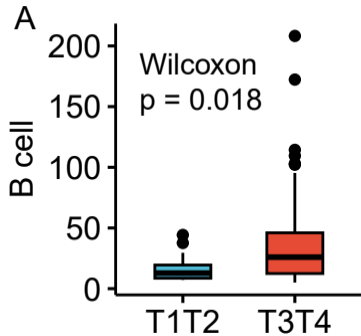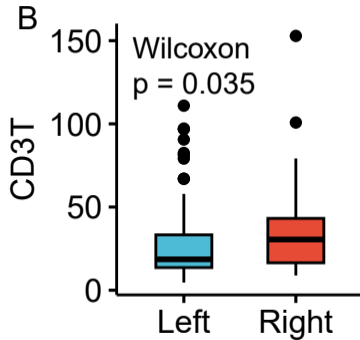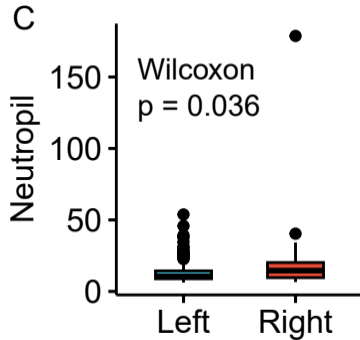

Supplement: Supplementary file 4 — Additional file 4 Figure S4 Distribution of B cell (A), CD3T cell (B) and neutrophil cell (C) according to T stages (A) and tumor location (B-C). The Y-axis represents the nearest distance to cancer cells [file 12967_2024_5418_MOESM4_ESM.pdf]

A

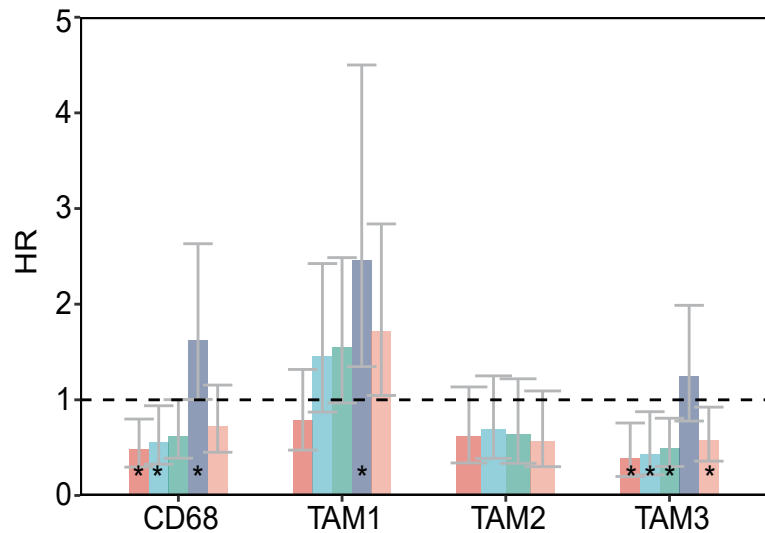

B

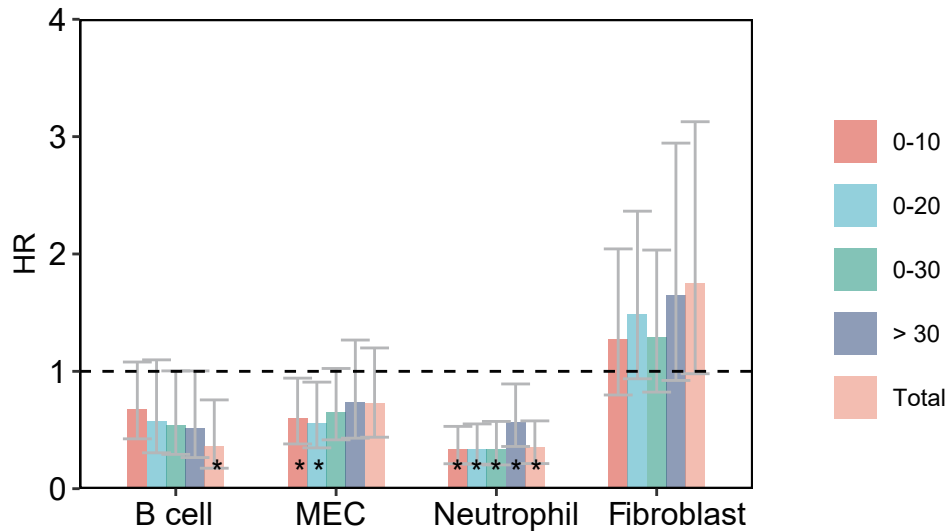

Supplement: Supplementary file 5 — Additional file 5 Figure S5. Bar plot showing the HR result for the M-panel–(A) and O-panel–(B) derived immune phenotypes. Bar height represents the HR value. Error bar, ± 95% CI. P values less than 0.05 are indicated with a star [file 12967_2024_5418_MOESM5_ESM.pdf]
